# Dental Regulations in the British Isles

**Published:** 1903-01

**Authors:** Walter Harrison

**Affiliations:** L.D.S. (Eng.), D.M.D. (Harv.), Brighton, England


### DENTAL REGULATIONS IN THE BRITISH ISLES.[1]

BY WALTER HARRISON, L.D.S. (ENG.), D.M.D. (HARV.), BRIGHTON, ENGLAND.[2]

It is a little difficult to write upon a political subject to be of international interest, and as it occurred to me that many of the members might be in doubt as to the mode of registration in this country, I thought a few lines might prove of value.

After the Dentist's Bill became an act in 1878, the administration of the same was handed over to the Medical Council. This council is practically a parliament in itself, and consists of three groups of members,—those elected by the medical profession by vote, those chosen by the various universities, colleges of physicians, and colleges of surgeons, and those appointed by the Privy Council. The dental profession has never had a representative *direct.* Mr. Charles Tomes, M.A. (Oxon.), F.R.S., F.R.C.S., L.D.S., author of "Comparative Dental Anatomy," etc., one of our most learned and highly esteemed dental surgeons, was appointed by the Privy Council, so, although we have one of the most able men possible to plead our cause, we cannot say we elected him to the position.

---

[1] Read before the Harvard Dental Alumni Association, June 23, 1902.

[2] President-elect of the British Dental Association.

### REGISTRATION AS A STUDENT.

The applicant must present a certificate of having passed a recognized preliminary examination consisting of English, arithmetic, Euclid, algebra, and Latin

He must also present a certificate from a registered dentist stating that he (or she) has commenced his study in dental mechanics, or from a recognized teacher of one of the various dental schools.

The majority of students select a private practitioner to obtain the benefit of his experience; but recently most of the schools have instituted a proper course of study in dental mechanics, and we now watch with great interest which course of study will prove the more useful to the future dentist. Each system has its advocates, but those whose homes are in the smaller towns, where no school exists, would be at a great disadvantage in time and cost, and most young men commence their studies at the age of sixteen or seventeen.

The course of study for the L.D.S. at all the colleges of surgeons is practically the same, and the period is identical,—viz., four years. Three years must be spent in acquiring the details of mechanical dentistry, and two years operative dentistry and general surgical and medical knowledge, making in all five years, although the authorities only require four. Those who reside in cities where a school exists can, of course, arrange their studies in the four.

### REGISTRATION AS A DENTIST.

Having attended all the lectures, demonstration hospital practice, and passed the various class examinations to the satisfaction of the staff of the dental school, the candidate presents himself at one of the colleges, and when he has satisfied the examiners of that body he obtains the diploma of L.D.S. (the examination is divided into three parts; see various Calendars), Licentiate of Dental Surgery. On presenting that diploma at the office of the Medical Council, and on payment of the fee of £5 his name is placed on the Dentists' Register. (See clause, Dentist's Act, on front of Register.)

The following are the authorities that grant the L.D.S.: Royal College of Surgeons, England; Royal College of Surgeons, Edinburgh; Royal College of Surgeons, Ireland; Royal College of Surgeons, Dublin; Faculty of Physicians and Surgeons, Glasgow.

The University of Birmingham, the youngest of our universities, and based upon American lines, grants degrees in dental surgery, B.D.S., M.D.S., but these degrees do not admit of registration at present. One is waiting to see what the value of the degree may be. A fair percentage of dentists take up what is called the conjoint diploma, M.R.C.S. and L.R.C.P., and a few a degree in medicine and surgery which can be added to the Dentists' Register, five shillings, as an additional qualification.

A registered medical practitioner can practise dental surgery, but cannot be registered in the Dentists' Register unless he possess the L.D.S. diploma. The conditions to qualify for such are the same as for dental students, with the exception that he is required to spend *two* years in the study of dental mechanics.

I have enclosed a copy of the Dental Register and also the various schools which contain the requirements of the various colleges of surgeons.

The L.D.S. diploma includes what you demand as the State examination, which I think a decided advantage over the American system, making one set of examinations to include the British empire.

American diplomas are not now registered by the Medical Council.

There is a slight contention between the Medical Council and some of the colleges as to the recognition of various schools (each claiming the right to power) for chemical and biological study at local technical schools.

The Medical Council has exercised the power of removing names from the register for unprofessional conduct, but the working of the act is outside the limit of these notes.

